# Supplementary material for: Serum bridging molecules drive candidal invasion of human but not mouse endothelial cells
Source: PLoS Pathog. 2022 Jul 7;18(7):e1010681. doi: 10.1371/journal.ppat.1010681 (PMC9295963; doi:10.1371/journal.ppat.1010681)
Supplement: S1 Fig — (A and B) Endocytosis (A) and cell-association (B) of live cells of a C. albicans egf1Δ/Δ cph1Δ/Δ mutant by human umbilical vein endothelial cells. (C and D) Effects of fresh and heat-inactivated serum on the endocytosis (C) and cell-association of the indicated strains of live C. glabrata. (E and F) Effects of fresh human serum and plasma on the endocytosis (E) and cell-association (F) of live C. glabrata. (G and H) Endocytosis (G) and cell-association (H) of live C. glabrata and S. cerevisiae. Results are the mean ± SD of 3 independent experiments, each performed in triplicate. Orgs/HPF, organisms per high-power field; ns, not significant; **P < 0.01, ***P < 0.001, ****P < 0.0001 by Student’s t-test (A and B) or ANOVA with the Dunnett’s test for multiple comparisons (C-H). (PDF) [file ppat.1010681.s001.pdf]

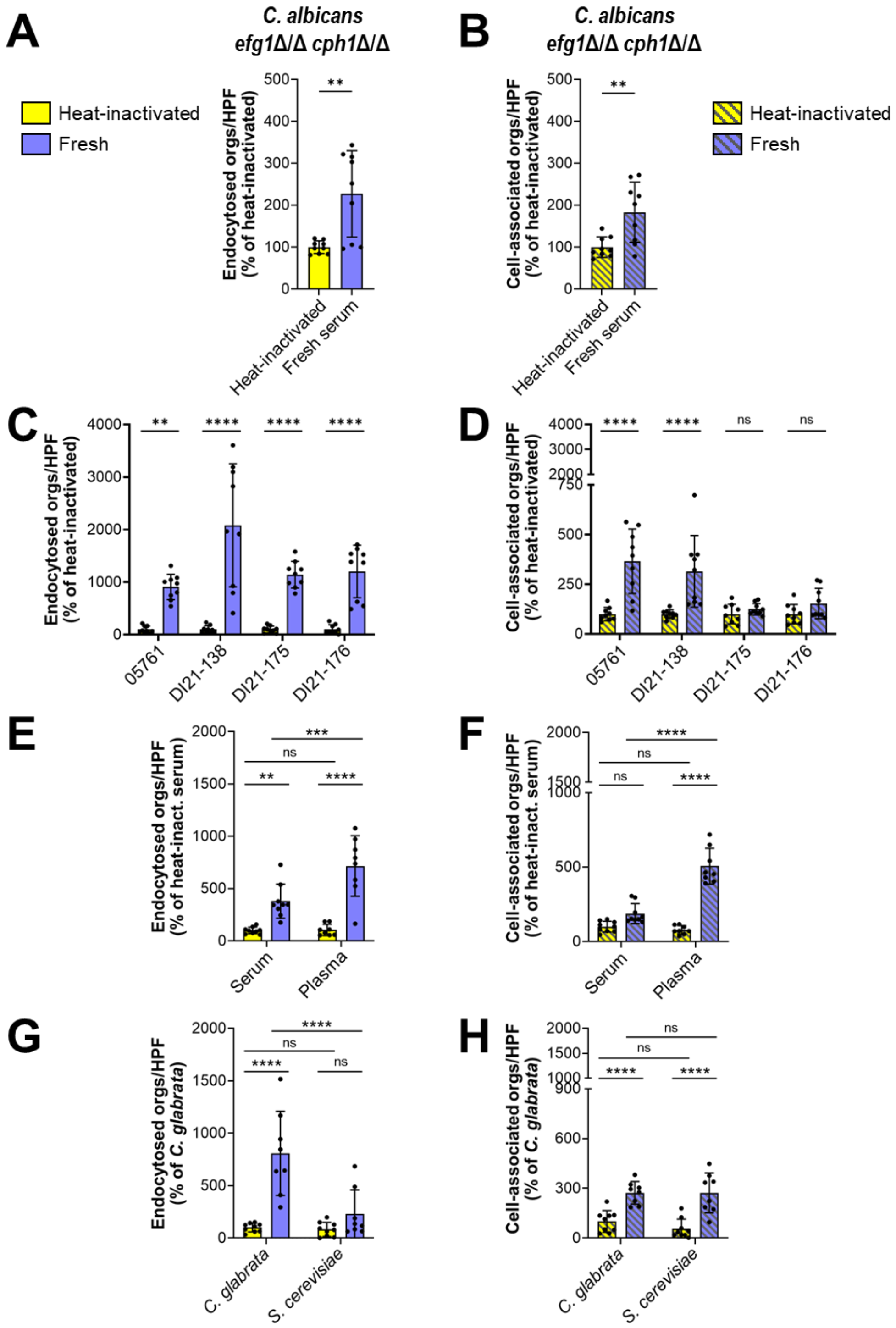

Fig. S1. Bridging molecules mediate the endocytosis of live *C. albicans* and *C. glabrata* yeast, but not *S. cerevisiae.* (A and B) Endocytosis (A) and cell-association (B) of live cells of a *C. albicans egf1*Δ/Δ *cph1*Δ/Δ mutant by human umbilical vein endothelial cells. (C and D) Effects of fresh and heat-inactivated serum on the endocytosis (C) and cell-association of the indicated strains of live *C. glabrata.* (E and F) Effects of fresh human serum and plasma on the endocytosis (E) and cell-association (F) of live *C. glabrata*. (G and H) Endocytosis (G) and cell-association (H) of live *C. glabrata* and *S. cerevisiae.* Results are the mean ± SD of 3 independent experiments, each performed in triplicate. Orgs/HPF, organisms per high-power field; ns, not significant; $^{**}P < 0.01$, $^{***}P < 0.001$, $^{****}P < 0.0001$ by Student's t-test (A and B) or ANOVA with the Dunnett's test for multiple comparisons (C-H).
