# Supplementary material for: Serum bridging molecules drive candidal invasion of human but not mouse endothelial cells
Source: PLoS Pathog. 2022 Jul 7;18(7):e1010681. doi: 10.1371/journal.ppat.1010681 (PMC9295963; doi:10.1371/journal.ppat.1010681)
Supplement: S2 Fig — (PDF) [file ppat.1010681.s002.pdf]

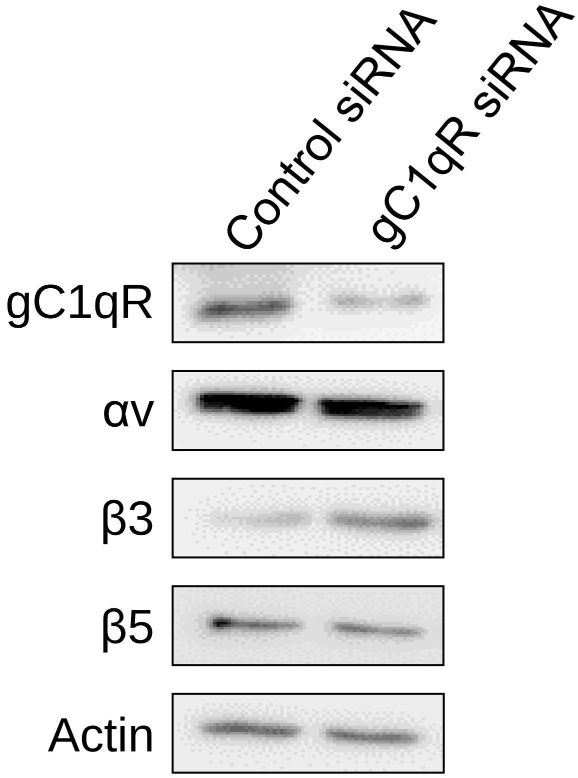

Fig. S2. Western blot showing effects of gC1qR siRNA on the levels of the indicated human endothelial cell proteins.
