# Supplementary material for: Serum bridging molecules drive candidal invasion of human but not mouse endothelial cells
Source: PLoS Pathog. 2022 Jul 7;18(7):e1010681. doi: 10.1371/journal.ppat.1010681 (PMC9295963; doi:10.1371/journal.ppat.1010681)
Supplement: S3 Fig — (A) Western blot showing effects of integrin αv siRNA on the levels of the indicated endothelial cell proteins. (B) Antibodies against gC1qR, integrin αvβ3, and integrin αvβ5 do not bind to C. glabrata. Flow cytometric analysis of C. glabrata cells that were incubated with antibodies against gC1qR (clone 74.5.2), integrin αvβ3, and integrin αvβ5. They were also incubated with control IgG and with a polyclonal anti-Candida antibody. Each histogram shows the analysis of 104 cells. (PDF) [file ppat.1010681.s003.pdf]

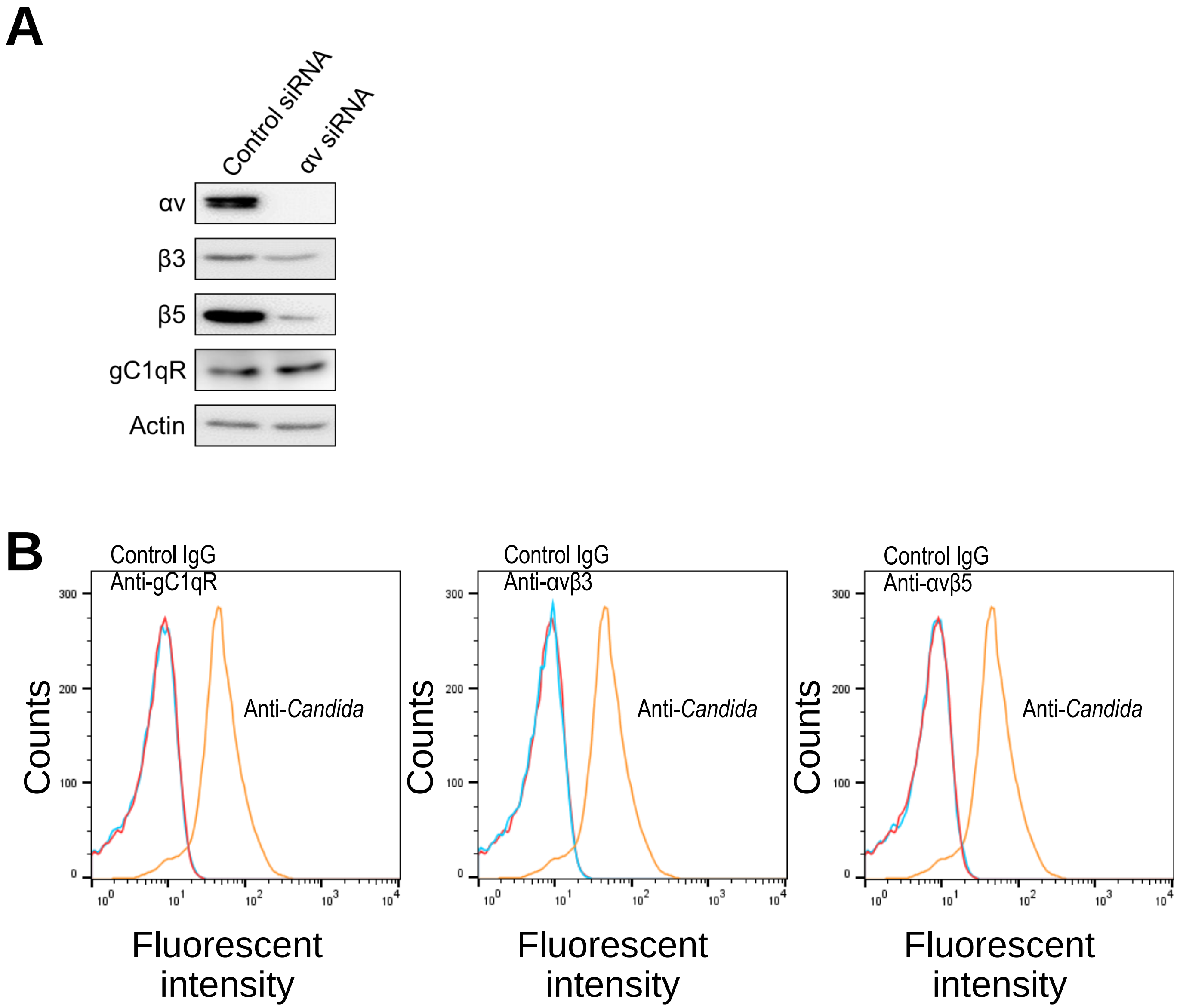

Fig. S3. (A) Western blot showing effects of integrin αv siRNA on the levels of the indicated endothelial cell proteins. (B) Antibodies against gC1qR, integrin αvβ3, and integrin αvβ5 do not bind to *C. glabrata.* Flow cytometric analysis of *C. glabrata* cells that were incubated with antibodies against gC1qR (clone 74.5.2), integrin αvβ3, and integrin αvβ5. They were also incubated with control IgG and with a polyclonal anti-*Candida* antibody. Each histogram shows the analysis of 10^4 cells.
