# Supplementary material for: Serum bridging molecules drive candidal invasion of human but not mouse endothelial cells
Source: PLoS Pathog. 2022 Jul 7;18(7):e1010681. doi: 10.1371/journal.ppat.1010681 (PMC9295963; doi:10.1371/journal.ppat.1010681)
Supplement: S4 Fig — (A and B) Combined effects of antibodies against gC1qR, integrin αvβ3, and integrin αvβ5 on the endocytosis (A) and cell association (B) of C. glabrata. Data are the mean ± SD of 3 experiments each performed in triplicate. HI, heat-inactivated; Orgs/HPF, organisms per high power field; ns, not significant; **P < 0.01; ****P < 0.0001 by ANOVA with the Dunnett’s test for multiple comparisons. (PDF) [file ppat.1010681.s004.pdf]

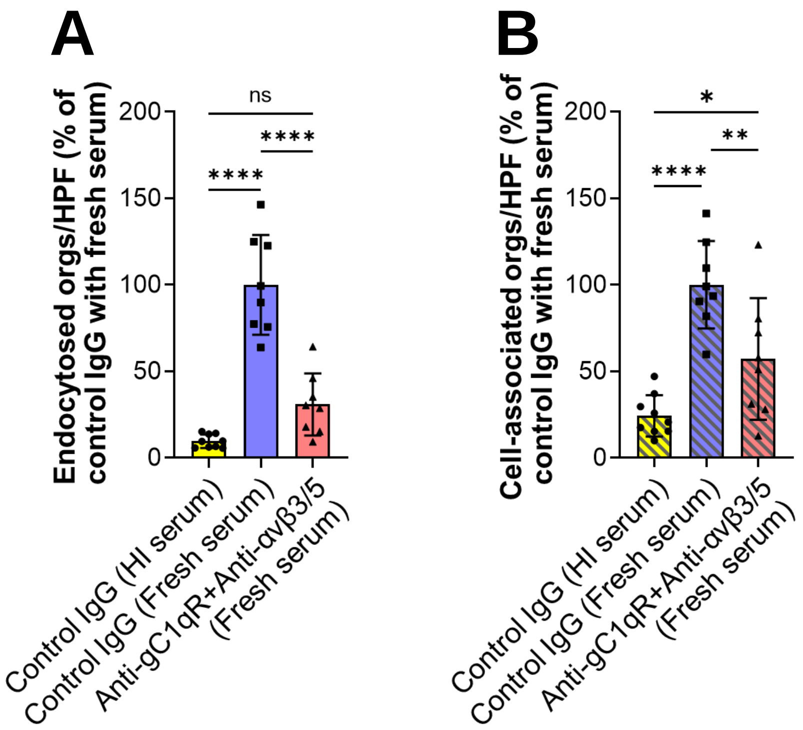

Fig. S4. (A and B) Combined effects of antibodies against gC1qR, integrin αvβ3, and integrin αvβ5 on the endocytosis (A) and cell association (B) of *C. glabrata*. Data are the mean ± SD of 3 experiments each performed in triplicate. HI, heat-inactivated; Orgs/HPF, organisms per high power field; ns, not significant; ***P* < 0.01; *****P* < 0.0001 by ANOVA with the Dunnett's test for multiple comparisons.
