# Supplementary material for: Serum bridging molecules drive candidal invasion of human but not mouse endothelial cells
Source: PLoS Pathog. 2022 Jul 7;18(7):e1010681. doi: 10.1371/journal.ppat.1010681 (PMC9295963; doi:10.1371/journal.ppat.1010681)
Supplement: S5 Fig — (A) Effects of antibodies against kininogen and vitronectin on the number of C. glabrata cells that were cell-associated with human endothelial cells. (B) Cell-association of the indicated number of C. glabrata cells that had been coated with either BSA or kininogen and vitronectin. (C) Effects of coating C. glabrata with BSA, kininogen, and/or vitronectin on the number of cell-associated organisms. (D) Flow cytometric detection of the binding of kininogen (top row) and vitronectin (bottom row) to C. albicans cells that had been incubated for 1 h with BSA alone, or with kininogen and vitronectin. Numbers in the upper right-hand corner indicate the percentage of positive cells. Results are representative of 4 separate experiments, each of which analyzed 10,000 cells. (E-F) Summary of combined flow cytometry results showing the binding of kininogen (E) and vitronectin (D) to C. albicans cells. Data in (A-C) are the mean ± SD of 3 experiments each performed in triplicate. Orgs/HPF, organisms per high power field; ns, not significant; ***P < 0.001; ****P < 0.0001 by ANOVA with the Dunnett’s test for multiple comparisons (A-C) or the Student’s t-test (E and F). (PDF) [file ppat.1010681.s005.pdf]

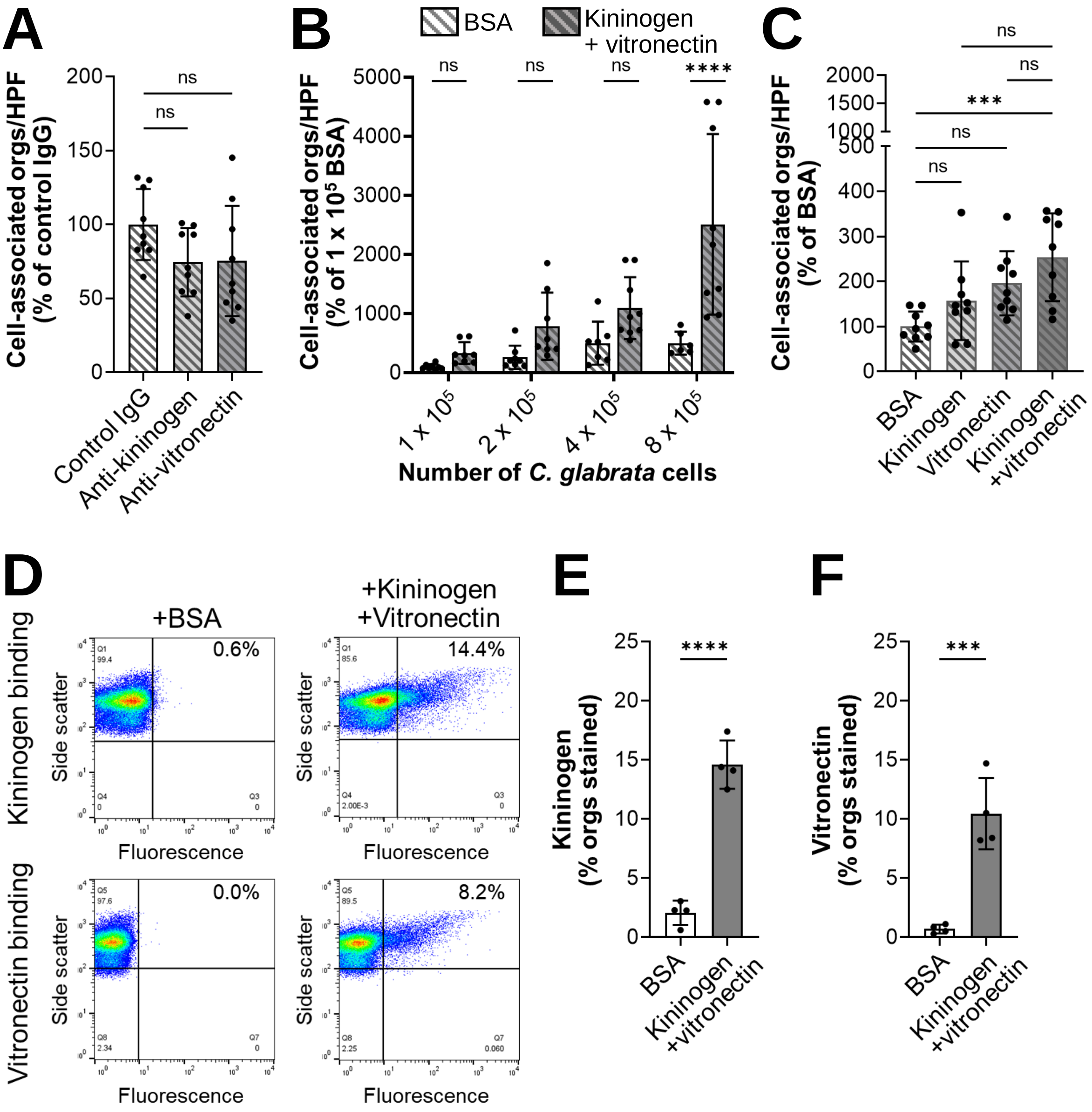

Fig. S5. (A) Effects of antibodies against kininogen and vitronectin on the number of cell-associated *C. glabrata* cells. (B) Cell-association of the indicated number of C. glabrata cells that had been coated with either BSA or kininogen and vitronectin. (C) Effects of coating *C. glabrata* with BSA, kininogen, and/or vitronectin on the number of cell-associated organisms. (D) Flow cytometric detection of the binding of kininogen (top row) and vitronectin (bottom row) to *C. albicans* cells that had been incubated for 1 h with BSA alone, or with kininogen and vitronectin. Numbers in the upper right-hand corner indicate the percentage of positive cells. Results are representative of 4 separate experiments, each of which analyzed 10,000 cells. (E-F) Summary of combined flow cytometry results showing the binding of kininogen (E) and vitronectin (D) to *C. albicans* cells. Data in (A-C) are the mean ± SD of 3 experiments each performed in triplicate. Orgs/HPF, organisms per high power field; ns, not significant; ***$P < 0.001$; ****$P < 0.0001$ by ANOVA with the Dunnett's test for multiple comparisons (A-C) or the Student's t-test (E and F).
