# Supplementary material for: Serum bridging molecules drive candidal invasion of human but not mouse endothelial cells
Source: PLoS Pathog. 2022 Jul 7;18(7):e1010681. doi: 10.1371/journal.ppat.1010681 (PMC9295963; doi:10.1371/journal.ppat.1010681)
Supplement: S6 Fig — (A and B) Effects of BSA or kininogen and vitronectin on the endocytosis of the indicated strains of C. albicans. (C) Kininogen and vitronectin increase cell-association (adherence) of the indicated Candida spp. (D) Endothelial cell damage caused by the cells of the indicated strains that had been coated with kininogen and vitronectin. (E) Kininogen and vitronectin do not enhance the cell-association of S. cerevisiae. (F and G) Inhibition of cell-association of C. glabrata coated with either vitronectin alone (F) or vitronectin and kininogen (G) by antibodies against gC1qR (clone 74.5.2) and/or integrins αvβ3 and αvβ5. Data are the mean ± SD of 3 experiments each performed in triplicate. Orgs/HPF, organisms per high power field; ns, not significant; *P < 0.05, **P < 0.01, ***P < 0.001, ****P < 0.0001 by Student’s t-test (A) or ANOVA with the Dunnett’s test for multiple comparisons (B-G). (PDF) [file ppat.1010681.s006.pdf]

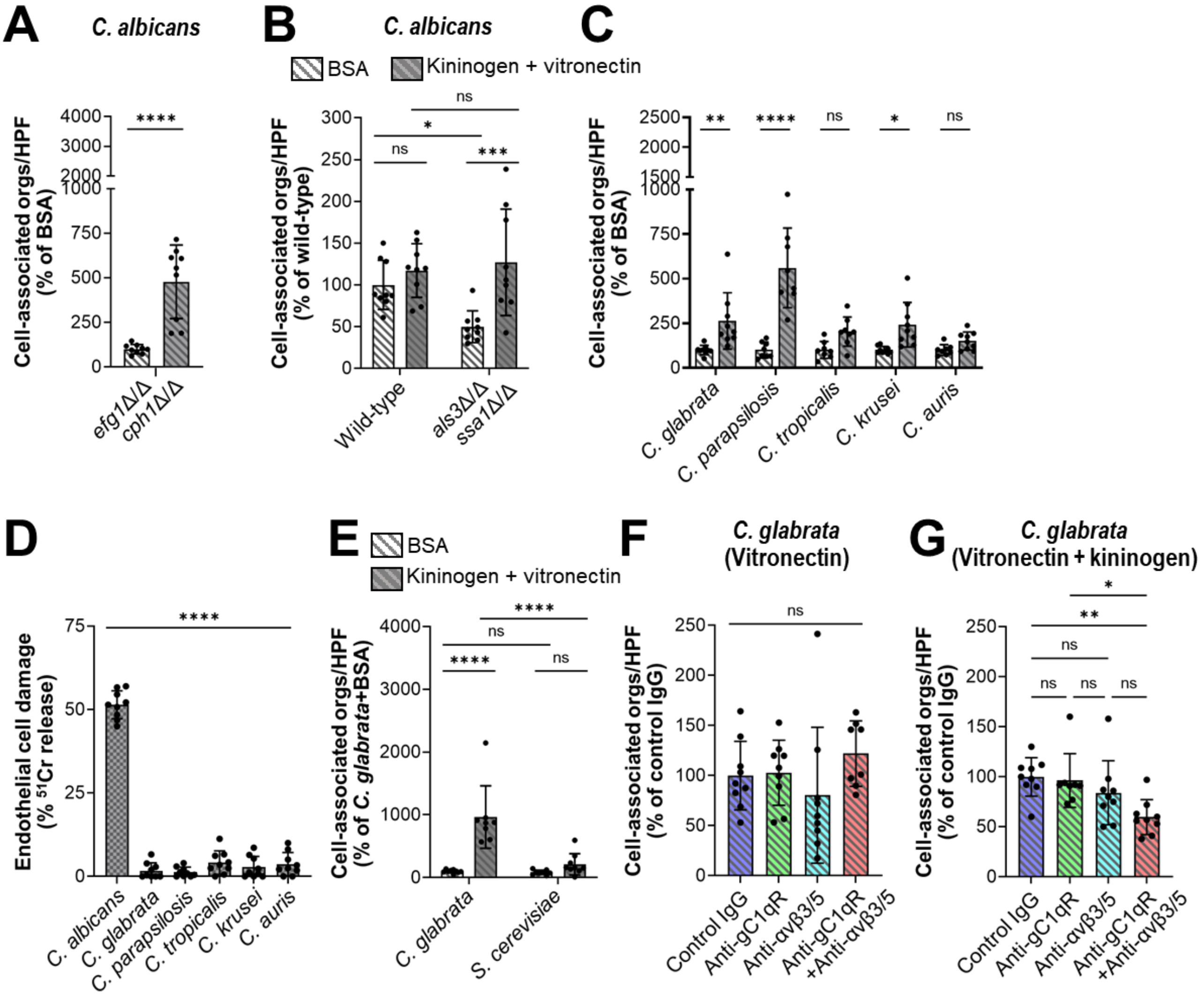

Fig. S6. Kininogen and vitronectin interact with gC1qR and αv integrins to induce adherence. (A and B) Effects of BSA or kininogen and vitronectin on the endocytosis of the indicated strains of *C. albicans*. (C) Kininogen and vitronectin increase cell-association (adherence) of the indicated *Candida spp.* (D) Endothelial cell damage caused by the cells of the indicated strains that had been coated with kininogen and vitronectin. (E) Kininogen and vitronectin do not enhance the cell-association of *S. cerevisiae.* (F and G) Inhibition of cell-association of *C. glabrata* coated with either vitronectin alone (F) or vitronectin and kininogen (G) by antibodies against gC1qR (clone 74.5.2) and/or integrins αvβ3 and αvβ5. Data are the mean ± SD of 3 experiments each performed in triplicate. Orgs/HPF, organisms per high power field; ns, not significant; *$P < 0.05$, **$P < 0.01$, ***$P < 0.001$, ****$P < 0.0001$ by Student's t-test (A) or ANOVA with the Dunnett's test for multiple comparisons (B-G).
