# Supplementary material for: Serum bridging molecules drive candidal invasion of human but not mouse endothelial cells
Source: PLoS Pathog. 2022 Jul 7;18(7):e1010681. doi: 10.1371/journal.ppat.1010681 (PMC9295963; doi:10.1371/journal.ppat.1010681)
Supplement: S7 Fig — (A and B) Cell-association of C. glabrata coated with either human or mouse serum by the indicated endothelial cells after 45 min (A) and 180 min (B). (C-F) Endocytosis (C and E) and cell-association (D and F) of C. glabrata coated with fresh human serum (C and D) or kininogen and vitronectin (E and F) by the indicated endothelial cells. (G) Western blot showing the protein levels of gC1qR, integrin αv and integrin β5 in mouse liver endothelial cells transduced with lentivirus containing the indicated human genes. gC1qR was detected with monoclonal antibody 60.1l, which only binds to the human protein. The integrins were detected with antibodies that recognize both human and mouse proteins. (H) Cell-association of C. glabrata coated with fresh human serum by mouse liver endothelial cells expressing human gC1qR, integrin αv, or integrin β5. Data in (A-F and H) are the mean ± SD of 3 experiments each performed in triplicate. HUVEC, human umbilical vein endothelial cell; orgs/HPF, organisms per high power field; ns, not significant; TIME, Tert-immortalized microvascular endothelial cells; *P < 0.05, **P < 0.01, ***P < 0.001, ****P < 0.0001 by ANOVA with the Dunnett’s test for multiple comparisons (A, B and H) or the Student’s t-test (C-F). (PDF) [file ppat.1010681.s007.pdf]

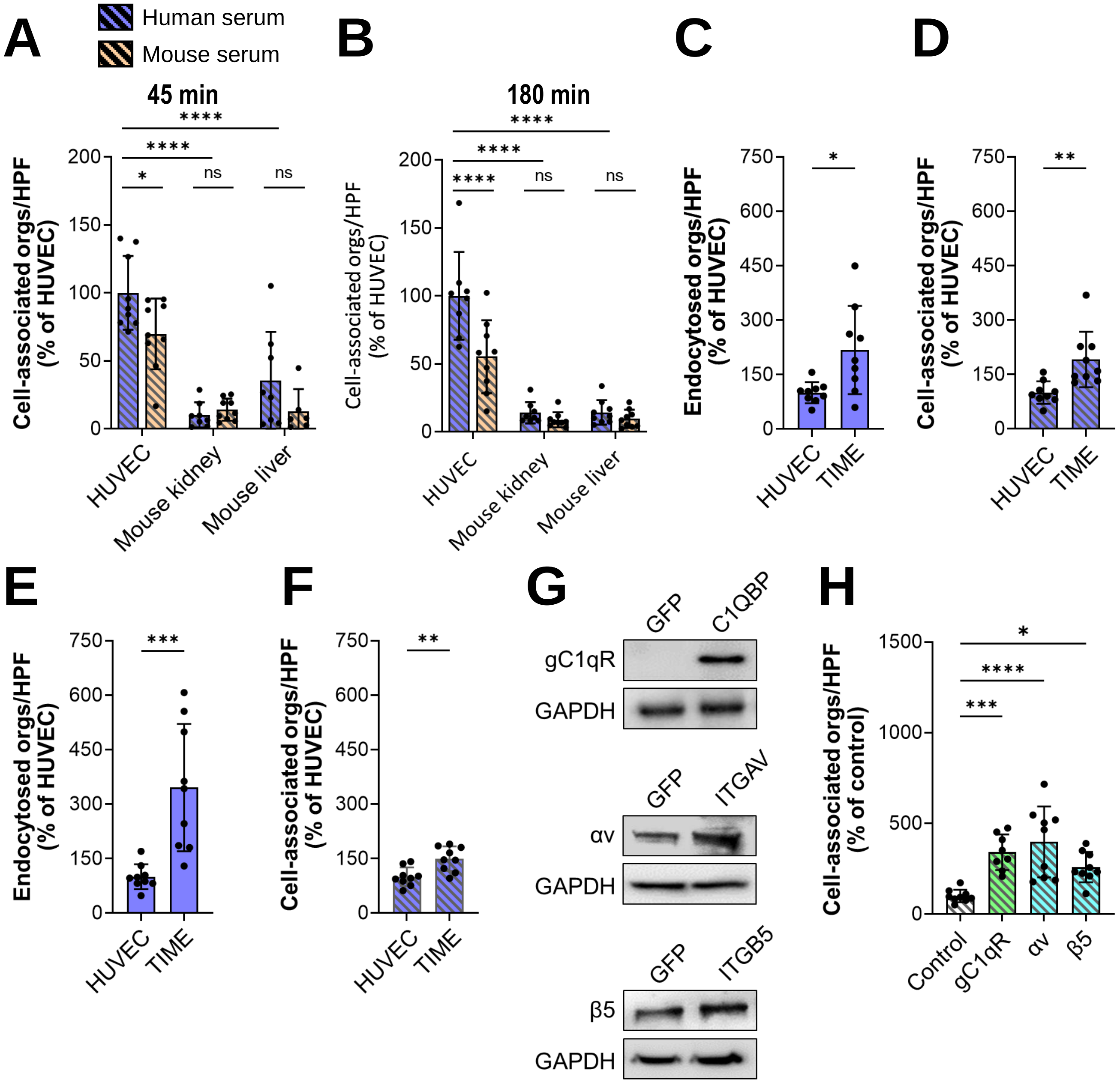


**Fig. S7.** Mouse endothelial cells poorly support bridging molecule-mediated adherence. (A and B) Cell-association of *C. glabrata* coated with either human or mouse serum by the indicated endothelial cells after 45 min (A) and 180 min (B). (C-F) Endocytosis (C and E) and cell-association (D and F) of *C. glabrata* coated with fresh human serum (C and D) or kininogen and vitronectin (E and F) by the indicated endothelial cells. (G) Western blot showing the protein levels of gC1qR, integrin αv and integrin β5 in mouse liver endothelial cells transduced with lentivirus containing the indicated human genes. gC1qR was detected with monoclonal antibody 60.1l, which only binds to the human protein. The integrins were detected with antibodies that recognize both human and mouse proteins. (H) Cell-association of *C. glabrata* coated with fresh human serum by mouse liver endothelial cells expressing human gC1qR, integrin αv, or integrin β5. Data in (A-F and H) are the mean ± SD of 3 experiments each performed in triplicate. HUVEC, human umbilical vein endothelial cell; orgs/HPF, organisms per high power field; ns, not significant; TIME, Tert-immortalized microvascular endothelial cells; *$P$ < 0.05, **$P$ < 0.01, ***$P$ < 0.001, ****$P$ < 0.0001 by ANOVA with the Dunnett's test for multiple comparisons (A, B and H) or the Student's t-test (C-F).
