# Supplementary material for: Serum bridging molecules drive candidal invasion of human but not mouse endothelial cells
Source: PLoS Pathog. 2022 Jul 7;18(7):e1010681. doi: 10.1371/journal.ppat.1010681 (PMC9295963; doi:10.1371/journal.ppat.1010681)
Supplement: S1 Table — (PDF) [file ppat.1010681.s008.pdf]

Supplementary Table 1. Fungal strains used in this work.

Organism	Strain	Relevant Genotype	Source/ Reference
Candida albicans	SC5314	Wild-type	[1]
Candida albicans	DIC185	*ura3*Δ::λ*imm434/ura3*Δ::λ*imm434*::*URA3-IRO1*<br>*arg4*::*hisG*/*arg4*::*hisG*::p*ARG4*<br>*his1*::*hisG/his1*::*hisG*::p*HIS1*	[2]
Candida albicans	CAI4-URA	*ura3*Δ*::imm434/ura3*Δ*::imm434::URA3*	[3]
Candida albicans	DSC10	*efg1*Δ*::hisG/efg1*Δ*::hisG*<br>*ura3*Δ*::* λ*imm434/ura3*Δ*::* λ*imm434::URA3-IRO1*	This study
Candida albicans	*ssa1*Δ/Δ-*als3*Δ/Δ	*ura3*Δ::λ*imm434/ura3*Δ::λ*imm434*<br>*ssa1*Δ::*FRT/ssa1*Δ::*FRT*<br>*SSA2/ssa2*Δ::*FRT*<br>*als3*Δ/*als3*Δ*::NAT1*<br>*RPS10/rps10::URA3*	[4]
Candida auris	CAU-09	Wild-type	[5]
Candida glabrata	05-761	Wild-type	[6]
Candida glabrata	DI121-138	Wild-type	Fungus Testing Laboratory at the University of Texas Health Science Center at San Antonio
Candida glabrata	DI121-175	Wild-type	Fungus Testing Laboratory at the University of Texas Health Science Center at San Antonio
Candida glabrata	DI121-176	Wild-type	Fungus Testing Laboratory at the University of Texas Health Science Center at San Antonio
Candida krusei	2333	Wild-type	Fungus Testing Laboratory at the University of Texas Health Science Center at San Antonio
Candida parapsilosis	1184	Wild-type	Fungus Testing Laboratory at the University of Texas Health Science Center at San Antonio

Candida tropicalis	DI13-280	Wild-type	Fungus Testing Laboratory at the University of Texas Health Science Center at San Antonio
Saccharomyces cerevisiae	AMP188	*MAT*α *lys-23 ade2 ura2-49* (Σ1278b background)	[7]

References

1. Jones T, Federspiel NA, Chibana H, Dungan J, Kalman S, Magee BB, et al. The diploid genome sequence of *Candida albicans*. Proc Natl Acad Sci USA. 2004;101(19):7329-34. PubMed PMID: 15123810.

2. Phan QT, Eng DK, Mostowy S, Park H, Cossart P, Filler SG. Role of endothelial cell septin 7 in the endocytosis of *Candida albicans*. MBio. 2013;4(6):00542-13.

3. Sanchez AA, Johnston DA, Myers C, Edwards JE, Jr., Mitchell AP, Filler SG. Relationship between *Candida albicans* virulence during experimental hematogenously disseminated Infection and endothelial cell damage In vitro. Infect Immun. 2004;72(1):598-601. PubMed PMID: 14688143.

4. Sun JN, Solis NV, Phan QT, Bajwa JS, Kashleva H, Thompson A, et al. Host cell invasion and virulence mediated by *Candida albicans* Ssa1. PLoS Pathog. 2010;6(11):e1001181. PubMed PMID: 21085601.

5. Mamouei Z, Alqarihi A, Singh S, Xu S, Mansour MK, Ibrahim AS, et al. Alexidine dihydrochloride has broad-spectrum activities against diverse fungal pathogens. mSphere. 2018;3(5). Epub 2018/11/02. doi: 10.1128/mSphere.00539-18. PubMed PMID: 30381356; PubMed Central PMCID: PMCPMC6211222.

6. Wiederhold NP, Najvar LK, Bocanegra R, Molina D, Olivo M, Graybill JR. In vivo efficacy of anidulafungin and caspofungin against *Candida glabrata* and association with in vitro potency in the presence of sera. Antimicrob Agents Chemother. 2007;51(5):1616-20.

7. Mitchell AP, Magasanik B. Three regulatory systems control production of glutamine synthetase in *Saccharomyces cerevisiae*. Mol Cell Biol. 1984;4(12):2767-73. Epub 1984/12/01. doi: 10.1128/mcb.4.12.2767. PubMed PMID: 6152013; PubMed Central PMCID: PMCPMC369287.
