# Supplementary material for: Serum bridging molecules drive candidal invasion of human but not mouse endothelial cells
Source: PLoS Pathog. 2022 Jul 7;18(7):e1010681. doi: 10.1371/journal.ppat.1010681 (PMC9295963; doi:10.1371/journal.ppat.1010681)
Supplement: Attachment — Submitted filename: C. glabrata bridging molecule paper- rebuttal-pp.pdf [file ppat.1010681.s010.pdf]

Reviewer #1:

1) Relevance of the findings to disease is not appropriately qualified. The host environment during infection is complex, with many cell types on the host side and different nutritional environments for the fungus. The impact of this work would be enhanced by some implication of these mechanisms during infection. One potential method would be to express human receptors in mice and add human serum proteins, to test the idea that increased interaction with endothelial cells through bridging serum proteins affects virulence of C. glabrata. This issue can also be addressed by appropriately qualifying the results to indicate that interactions in the whole mouse or human could be quite different from interactions in culture (e.g. add "mouse endothelial cells in culture" to line 338; edit lines 348/349 to read "mouse and human endothelial cells in culture"; add "in vitro" to the end of the sentence in line 355 and include the suggestion that these processes may be more complex in vivo).

Response. We fully agree with this important point and have modified the text as suggested.

Reviewer #2: None

Reviewer #3: None identified.

Part III – Minor Issues: Editorial and Data Presentation Modifications

Reviewer #1: 2) There can be quite a bit of variation among strains in a given species, including C. albicans (Marakalala et al. 2013) and C. glabrata (Ost et al 2021). This should be more fully discussed in the Discussion, given that it appears that only one representative of each strain was assayed.

Comment. We agree that there can be significant strain-to-strain variation in *Candida* spp. To investigate this variation, we tested the effects of serum on 3 additional blood isolates of *C. glabrata.* As shown in Figs. S1C and D, fresh serum greatly enhanced the endocytosis of all of these strains, although it only increased the adherence of two of them.

Reviewer #2: Figure 1 Statistics: Verify the statistical analysis for 1 H and 1 I. Multiple comparison testing is listed, but it appears to only need single analysis.

Response. The reviewer is correct. The statistical analysis of the cytochalasin D data in Figs. 1H and I used the Student's t-test because there was only a single comparison. This has been corrected in the legend.

Figures 1 and 2 legend: Clarify that these are human endothelial cells.

Response. Done.

Figure 2 legend: Add statistical analysis description. For the antibodies, describe their different binding sites in the legend.

Response. Done.

Line 109: Add a brief description about the relevance of the different C- and N-terminus antibody results.

Response. Done.

Line 115: Were any negative results found (integrins that didn't bind)? Those could be included in supplementary data.

Response. We apologize that our prior description of the screen process was unclear. We actually tested the effects of siRNA knockdown of integrins αv and α5 and found that only knockdown of integrin αv reduced the endocytosis and adherence of serum coated *C. glabrata* (Fig. 3A and B). We then tested the effects of neutralizing antibodies against integrins αvB3 and αvβ5 and found that both of these antibodies inhibited the interactions of serum coated organisms with endothelial cells (Fig. 3C-F). We have revised the text to reflect this (lines 115-120).

Figure 3, 4, and 5 legend: Add that that the cells are human and add statistical analyses tests

Response. Done.

Figure 5 D and E: Describe colors in legend or add to figure

Response. The figure has been corrected.

Figure 7: Add statistical tests

Response. Done.

Supplementary figures: Add statistical tests used and that the endothelial cells were human where relevant.

Response. Done.

Figure S6D and E: add a description of the colors for the bars

Response. This was done for S6E. All bars in S6D are the same color.

Reviewer #3: 1. The authors mention that "Although...yeast-phase C. parapsilosis cells are endocytosed by endothelial cells in vitro, this process is much slower and less efficient than the endocytosis of hyphal-phase C. albicans (Shintaku et al.). A limitation of these previous experiments is that they were performed in serum-free media." This sentence does not accurately reflect the data presented in the Shintaku paper. In fact, C. parapsilosis yeast underwent endocytosis with considerably higher efficiency than C. albicans hyphae (Fig. 3c) and in agreement with the present work, serum was required (Fig. 3d). The authors should reconsider these prior results in the context/discussion of their experiments.

Response. We thank the reviewer for pointing this out. We have modified the introduction to mention that it is known that the endocytosis of yeast phase *C. parapsilosis* is enhanced by the presence of serum (lines 51-53). Because the studies of Shintaku et al used heat killed hyphae which are endocytosed much less efficiently than live hyphae (Sintaku et al. found no endocytosis of heat-killed hyphae until 24 h whereas we found avid endocytosis of live hyphae within 1.5 h (Fig. 5B)), we removed the comparison of *C. parapsilosis* to *C. albicans* hyphae.

2. The confocal micrographs in Fig. 3I are important data to support the role of gC1qR and avB5 as receptors for serum coated C. glabrata. Because the images with heat-inactivated serum are important to support the overall conclusions, it would be helpful if they were included in the same figure to facilitate side-by-side comparisons rather than in a supplemental figure.

Response. We have made the suggested changes.

3. Line 256 - The Western blot is actually Fig. S7G (not S7E)

Response. Corrected.

4. Line 260 - These data are depicted in Fig. S7H (not S7G)

Response. Corrected.
